# Dynamic Thinking Approach of Non-medical Use of Pharmaceutical Opioids

**DOI:** 10.22037/ijpr.2019.1100886

**Published:** 2019

**Authors:** Farahnaz Zeinali, Ali Rajabzadeh Ghatari, Mehdi Mohammadzadeh, Fatemeh Mojibian

**Affiliations:** a *Department of Phamaeconomics, School of Medicine, Shahid Beheshti University of Medical Sciences, Tehran, Iran. *; b *Department of Industrial Management, School of Management and Economics, Tarbiat Modares University,Tehran, Iran. *; c *Department of Pharmacoeconomics and Pharmaceutical Management, School of Medicine, Shahid Beheshti University of Medical Sciences, Tehran, Iran.*; d *Department of Industrial Management, School of Management and Economics, Tarbiat Modares University, Tehran, Iran.*

**Keywords:** System dynamics, Qualitative model, Causal loop diagrams, Pharmaceutical opioids, Addiction

## Abstract

Unsteady increase in using pharmaceutical opioids is a phenomenon that has existed in human societies for a long time. Furthermore, the ever-growing trend of addiction to opioids affects communities in various economic, social, and cultural aspects. Since abusing pharmaceutical opioids is a complex dynamic problem, it is extremely difficult to recognize the factors influencing this abuse. Thus, applying such dynamic models as system dynamics (SD) plays an important role in addressing these complex dynamic problems. System dynamics model falls in two categories of qualitative and quantitative system dynamics. In this paper, causal loop diagrams (CLDs), which are conceptual qualitative stages were applied, and variables were elicited from literatures; then, the initial CLDs were generated, and afterward, some experts were interviewed in order for the CLDs model to be evaluated. Ultimately, a meeting was held for discussing the variables and validating the diagrams. In this study, variables are connected by causal relationships called reinforcing and balancing. Then, the CLD model clearly depicted how the variables interact with one another indifferent stages of the process. The CLDs model is a fundamental issue in developing quantitative system dynamic, assisting policymakers in forecasting the process of non-medical use of pharmaceutical opioids and finding some measures to reduce their usage.

## Introduction

Abusing pharmaceutical opioids has been noticeably rising in human societies in recent years. In spite of the fact that public awareness has raised in this regard, this problem has expanded considerably in many countries. It is self-evident that addiction to pharmaceutical opioids influences societies in diverse economic, social, and cultural perspectives (1). A study in the USA showed that, from 1996 to 2011, misusing pharmaceutical opioids escalated; this research, however, indicated that the use of illicit drugs decreased in the same period (2). Researchers generally pay attention to the use of illicit drugs such as heroin and cocaine**, **but pharmaceutical opioids are usually ignored (3).

It is crystal clear that the growing use of pharmaceutical opioids by patients leads to increasing misuse, abuse, and diversion (4). Although pharmaceutical opioids are beneficial to relieving pain in malignancies such as cancers (5), during the last years, they have also been increasingly popular in relieving chronic non-cancer pain (6). For example, using pharmaceutical opioids may be rather a good idea for comforting patients.

However, it can cause some difficulties for them later (7). Using long-acting opioids in particular can increase their dependency and addiction, ending up in death.

According to reports, 2.1% of the population in the USA abused pharmaceutical opioids in 2009.Moreover, the mortality rate due to using opioids became threefold from 1999 to 2006 (8). In 2007, the Food and Drug Administration (FDA) implemented a strategy called “the risk evaluation and mitigation strategy” (REMS) (9),which is applied for the medicines that are effective but have serious risks. According to REMS, producers of these medicines should inform physicians of some information such as proper prescription and attention to opioids users’ misuse and dependency (10). Unfortunately, there is no experiment indicating that REMS is useful in reducing the use and abuse of pharmaceutical opioids (11).

Policy-makers’ efforts to reduce and manage the overuse and misuse of pharmaceutical opioids have been unsuccessful (11). Therefore, tools and interventions are required to address these problems and assist policy-makers in recognizing and solving them properly.

This paper aims to develop a tool named system dynamics as the integrating factors affecting the misuse of pharmaceutical opioids. System dynamics is a way to help researchers to realize the structure of the system and forecast the misleading results (12).

*System Dynamics Model*

A system is defined as a set of components working in a regular series. The variables of a linear approach in a system are connected linearly and in one way; however, in the systemic approach, the relations among these variables are not one-sided, but based on cause and effect (13). Unlike the linear approach, a complex system is a system whose behavior cannot be easily predicted. As we know, people tend to solve problems quickly. They consider their problems as a linear system and do not pay attention to delays in performance results (14). Hence, they cannot notice the interaction between the subjects, so they make mistakes in solving problems.

Due to the complexity and disturbance in the real world, decision-makers have problems in deciding and solving challenges. Thus, using a simulation system to overcome these disabilities is socially important.

System dynamics is a powerful tool to analyze and simulate cases. It was first developed by Forrester in the 1950s at MIT, but was gradually applied in other fields such as industry, economics, biology, and medical sciences (12). In some studies, system dynamics is employed in health care systems, including lymphocyte interactions (15), diagnose in diabetes population (16), pharmaceutical opioids overdose in chronic pain (17), and developing programs in healthcare (18).

Several structures for processing system dynamics have been defined. Generally, SD *consists *of four stages, including conceptualization, formulation, testing, and implementation (19). The first stage is the qualitative phase of SD, and the others are related to quantitative phase (20).

The qualitative phase defines problems and generates causal loop diagrams (CLD) (20). The relationship between the factors in a system is not visible; therefore, CLD is applied to demonstrate how a variable affects others (21). In CLD models, the relationship between variables is demonstrated by arrows (causal link). In the link between two variables, if the increase in a former variable leads to the increase in the latter, this link is named positive. Likewise, if the increase in one variable leads to the decrease in another one, this link is called negative. Since SD has a dynamic behavior, CLD shows feedback relationships in variables to help realize the dynamics in the structure of the system (22). The reinforcing feedback loops are called positive, and the balancing loops are called negative. CLD is designed to know the behavior and interaction between complex systems (12).

*Method*

In this study, the initial model of the factors affecting pharmaceutical opioids abuse was designed according to the system dynamics approach, literature, and experts’ opinions. This means that some CLD models for the relationships among the variables were proposed and evaluated. 

The factors cover eight categories, including pain control, access, trust in physicians, family roles, tendency to abuse, fear of abuse, regulations, and other ones. The aim of designing factors extracted from the literature was to generate initial causal loops (23).

After generating the initial CLD model, a mental model of experts was extracted based on their experience and knowledge (24). To do so, ten leading experts, who are well-qualified in the treatment of chronic diseases and addiction treatment, were invited. To have a more comprehensive view, some experts practicing in Iran Drug Control Headquarters and in Iranian National Center for Addiction Studies with at least 5 years of experience also participated in this phase of study. More specifically, these experts mentioned their opinions about the factors and their relationships through an interview. This means that the interviewees were asked to add or drop variables and check the missing variables in each loop. Finally, a meeting was held in order for us to discuss the CLD model and modify the diagram. In other words, the proposed CLD model was supposed to be validated by the experts who are involved in the use of pharmaceutical opioids. Thus, model validation is an essential part of the model development to ensure that the model defines this reality properly.

The following sections discuss the structure of each loop in order to develop the final conceptual qualitative model.

## Results

Since it is time-dependent, forecasting the abuse of pharmaceutical opioids is a dynamic process, and due to a variety of effective factors, it is a complex system.

The most required information for developing a CLD model is received by a conceptual model from the real world through contacting with the aforementioned experts (25). A number of reinforcing **(R)** and balancing **(B)** loops are recommended for both CLD models. The first model is about the abuse and diversion of pharmaceutical opioids in patients suffering from chronic pain such as cancers. The second one has to do with the addicted people who received diverted or smuggled pharmaceutical opioids. Having considered the data obtained from the interviews and discussions in meetings and comparing the experts’ opinions, the validated causal loop diagrams were developed. In other words, in this study, experts play key roles in developing and validating the CLD models.

**Figure 1:**
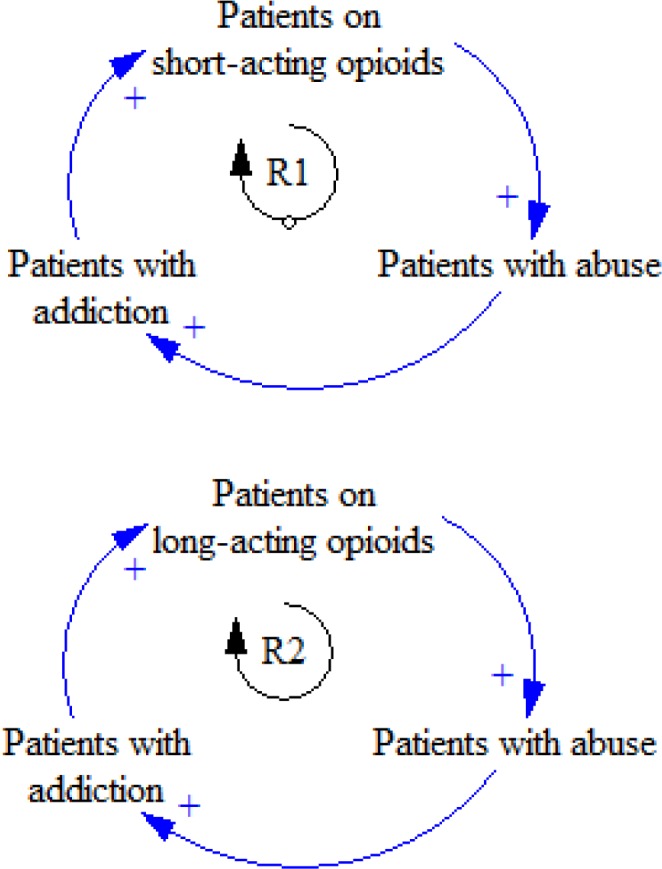
R1 and R2 feedback loops

Every year, patients suffering from chronic pain take opioids to reduce their pain; they are treated by short-acting or long-acting pharmaceutical opioids. Those patients whose pain is relieved may cut their medicine. It is shown that in similar frequent exposures to opioids, patients on long-acting opioids are exposed to a higher rate of abuse compared to those on short-acting opioids (26). If their pain gets severe, the patient may change taking short-acting opioids to long-acting ones. Every year, a number of patients taking short-acting or long-acting opioids are inclined to abuse opioids; thus, they may become addicted to them (Figure 1).

According to a study, the increase in patients’ addiction makes physicians pay much attention to prescribing opioids (27).

**Figure 2:**
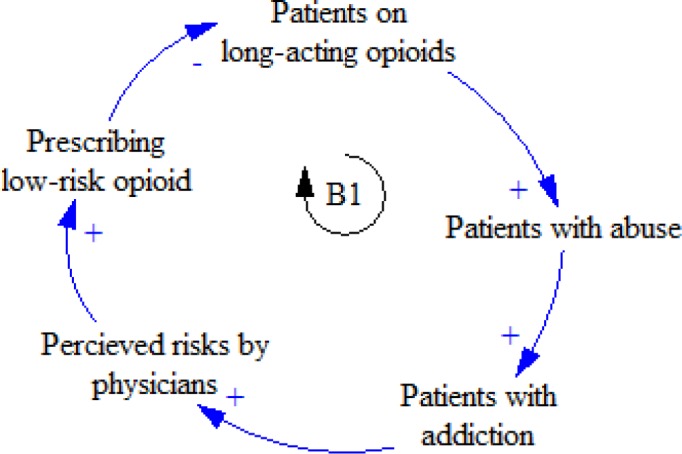
B1 feedback loop

Encountering the patients whose addiction to long-acting opioids increases, physicians begin to pay attention to grave risks and try to prescribe short-acting opioids to decrease the risks of patients’ addiction. (Figure 2).

**Figure 3:**
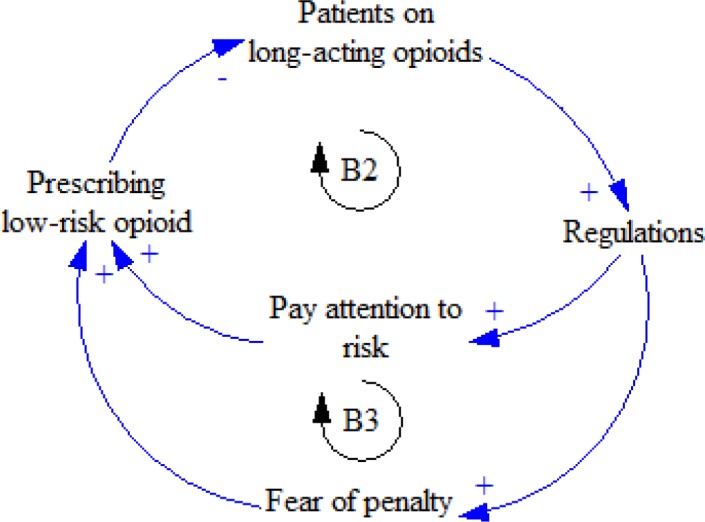
B2 and B3 feedback loops

In addition, since using long- acting opioids causes some difficulties such as drug abuse in a society, policy-makers make strict regulations for physicians in order to reduce the administration of these drugs, and physicians pay attention to the risks leading to abuse (28). Moreover, as these physicians fear penalty, they tend to administer low-risk drugs; consequently, the use of long- acting opioids will be reduced (Figure 3).

**Figure 4:**
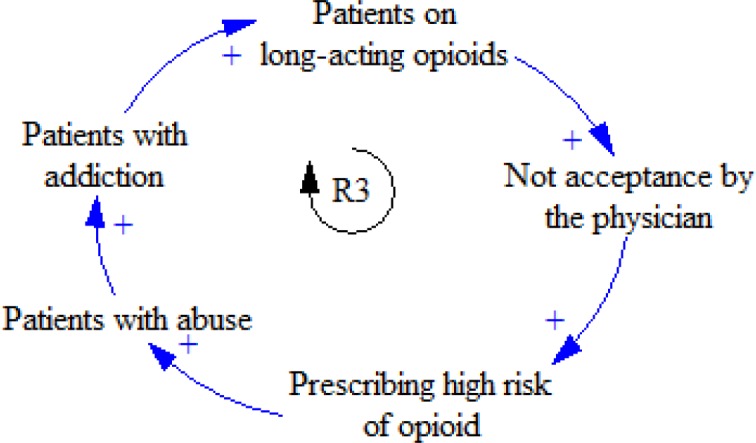
R3 feedback loop

Additionally, in some countries, the FDA has made guidelines to administrate drugs efficiently. Some physicians do not cooperate with the FDA and may increasingly administrate long-acting opioids for relieving their patients’ pain (Figure 4).

**Figure 5:**
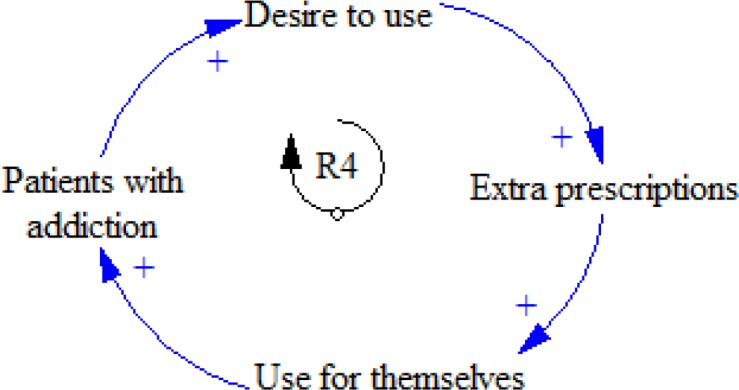
R4 feedback loop

The popularity of using opioids increases in the addicted patients who always follow the opportunities to prepare extra opioids. One of the ways of taking extra opioids is doctor shopping, which means “obtaining prescriptions from different physicians for the same pharmaceutical opioid”(29) which increase patients’ addiction (Figure 5).

According to some studies, surveying the diversion of the prescription of opioids, as the main agent of supply for abusing these kinds of drugs, is so important (30).

**Figure 6:**
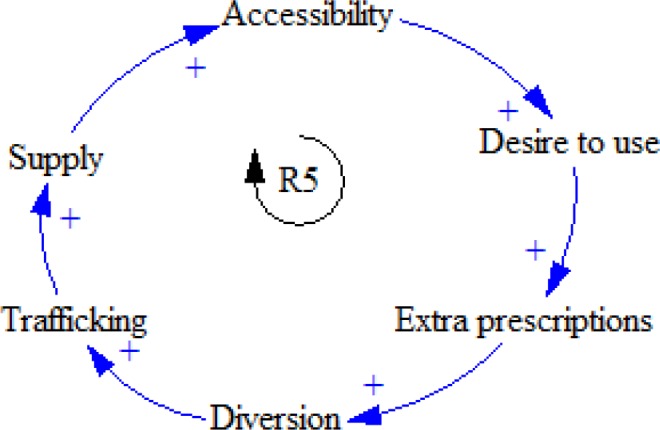
R5 feedback loop

A number of extra prescriptions may be diverted to addicted people rather than patients. Consequently, the amount of trafficking these opioids will increase, leading to boosting the supply and accessibility of opioids. By having more access to these opioids, people tend to use non-medical opioids more and more, which results in extra prescription (Figure 6).

**Figure 7:**
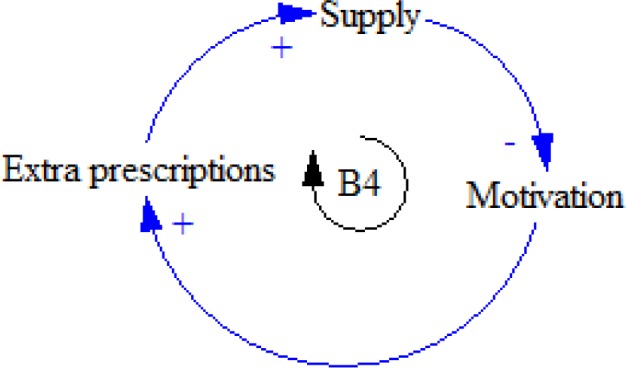
B4 feedback loop

When the supply of opioids is reduced due to the shortage of prescription (because of any reason), patients’ motivation for preparing extra prescription increases, as a result of which doctor-shopping appears (Figure 7).

Families play a key role in increasing or decreasing the abuse of pharmaceutical opioids. If the patient is related to an addicted person in the family or to his or her friends, the risk of abuse will rise.

**Figure 8:**
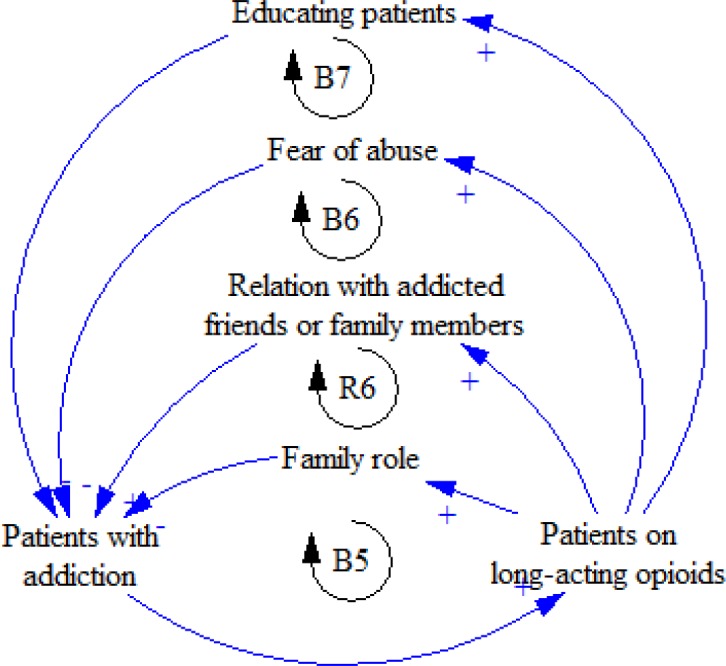
R6, B5, B6, and B7 feedback loops

Relatives’ concern and fear of addiction are two major factors that prevent the abuse; educating patients, also, helps reduce opioids abuse (Figure 8).

**Figure 9:**
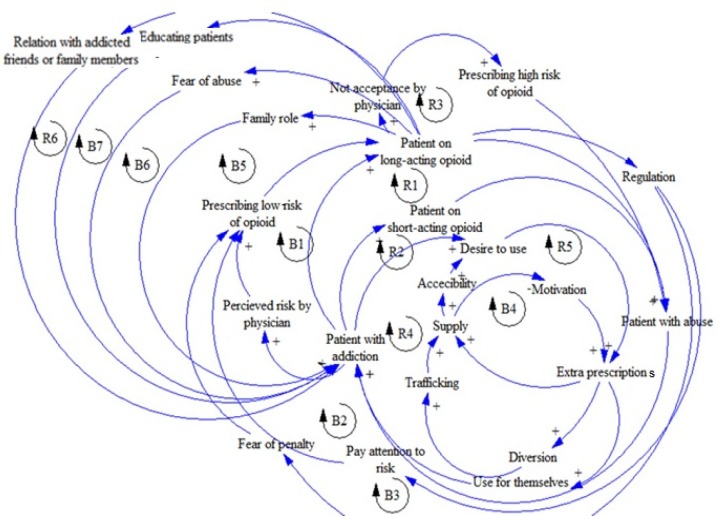
Causal loop diagram of use, abuse, and diversion of pharmaceutical opioids in patients suffering from chronic pain

Figure 9 shows the general validated causal loop diagram of use, abuse, and diversion of pharmaceutical opioids in patients, including reinforcing and balancing loops. As mentioned earlier, reinforcing loops play a strengthening role in the loops. Moreover, balancing loops acts as a controlling factor in the loops.

**Figure 10:**
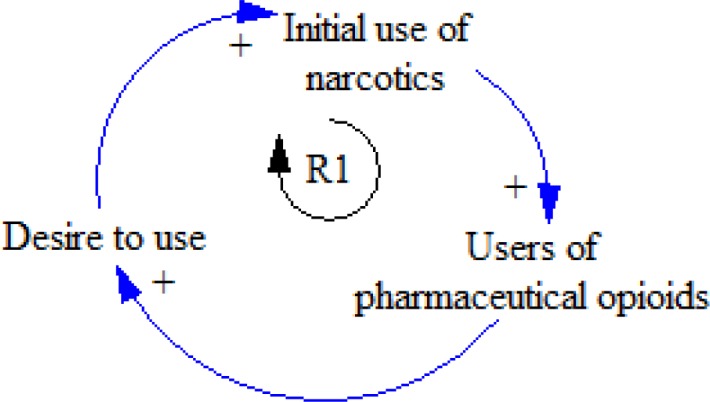
R1 feedback loops

**Figure 11:**
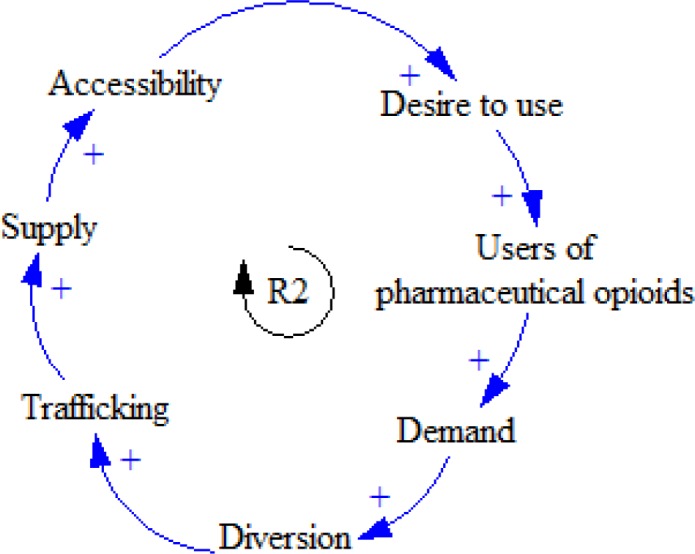
R2 feedback loop

**Figure 12:**
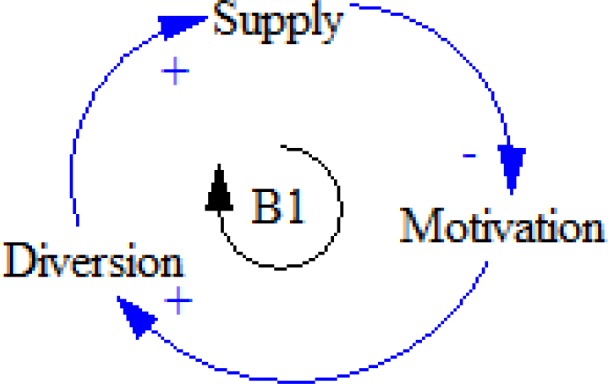
B1 feedback loop

**Figure 13:**
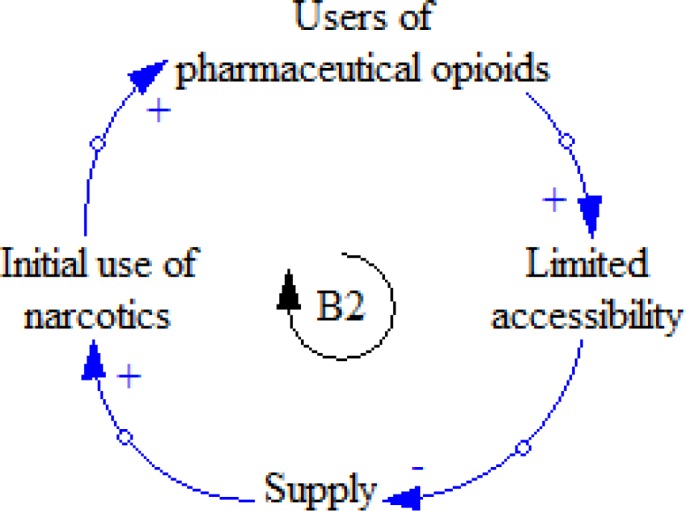
B2 feedback loop

**Figure 14:**
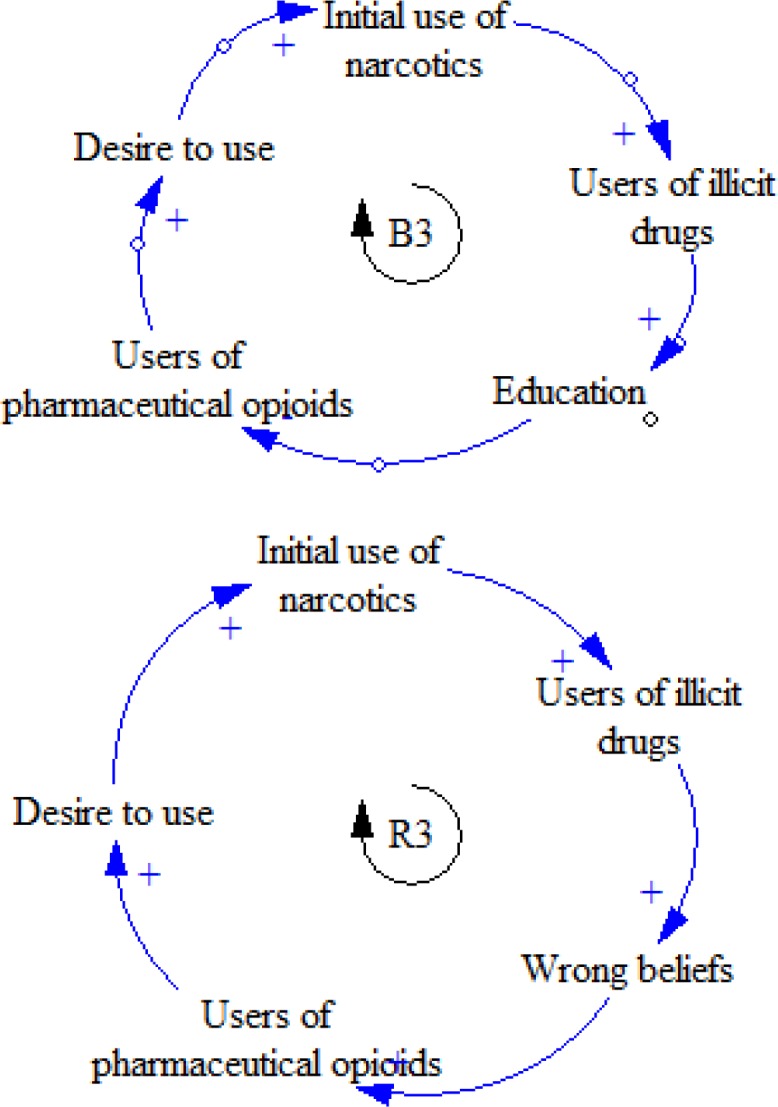
B1 and R3 causal loops

**Figure 15:**
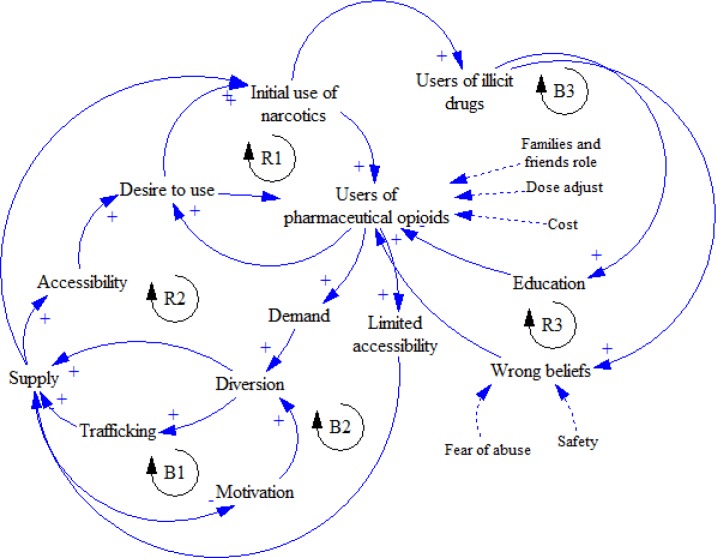
Causal loop diagram of pharmaceutical opioids abuses in addicted people

Every year, some people resort to narcotics. When the amount of using narcotics increases in a society, the number of people using illegal narcotics goes up. Some of these people may be inclined to use pharmaceutical opioids. Some of the pharmaceutical opioid abusers get free or stolen opioids from their family or friends who receive prescription from physicians. Others obtain opioids from dealers and smugglers (9). With the growing number of addicted people, non-addicted people become interested in narcotics as a result of their contact with these addicts. Therefore, the number of individuals who use narcotics for the first time increases (Figure 10).

It appears that some demands are via smuggling and the others via the diversion of opioids by the patients who receive them legally (31). According to the model, by increasing the demands, the diversion will be augmented, leading to a rise in trafficking, which, in turn, culminates in higher supply and accessibility (Figure 11).

Sometimes the number of prescriptions decreases for some reasons; therefore, motivation for diversion will grow. As a result, the patient who diverts tries to get more prescriptions to get more money (Figure 12).

As the number of non-medical users of pharmaceutical opioids increases, their diversion cannot meet their demands, so accessibility decreases, leading to reducing the initiation rate of use and users (Figure 13).

Some addicted people believe that the risks of using pharmaceutical opioids are fewer than those of narcotics. Educating these people and making them aware of having this wrong mentality ends up in reducing the popularity of using these opioids (Figure 14).

Figure 15 shows the general causal loop diagram of addicted people who use pharmaceutical opioids. It is reinforcing and balancing combination loops which affect each other.

## Conclusion

The aim of this paper was to explain the application of system thinking of the factors influencing the abuse of pharmaceutical opioids and to present a causal loop diagram methodology with the following three steps:

1. Extracting the factors from the literature and experts’ opinions and generating the initial causal loop diagram.
2. Using experts’ knowledge and experience by face-to-face interview to elicit a mental model for evaluating and completing CLD.
3. Holding a meeting in order to discuss the CLD model for modifying and validating the diagram.

It is worth mentioning that this study is just an exploratory one, trying to uncover influential variables and to validate proposed loops by inviting key informants in the area of abusing pharmaceutical opioids. With this in mind, most of the literature in this area has focused on using quantitative studies called stock and flow diagram (SFD), while the body of the literature addressing our approach to present causal loop diagrams (CLD) is dearth. Therefore, it is too difficult for authors to compare the results with those of other studies. However, in two studies, Wakeland *et al*. (2011) tried to find causal loops ending up to death, while, in this study, detecting such variables was not possible owing to lack of valid database(17, 32). As mentioned earlier, CLD model includes several variables connected by causal relationships; in this regard, according to our findings, some reinforcing and balancing loops were created. Variables in reinforcing loops kept increasing, while variables in balancing loops remained stable overtime. The CLD model is based on creating the quantitative system dynamics model, which assisted researchers in implementing and simulating the model by computer modeling tools. In addition, a qualitative and quantitative system dynamics permits stakeholders to predict the abuse process of pharmaceutical opioids and evaluate the potential interventions to reduce non-medical use of pharmaceutical opioids.

Consequently, the results of this study presented in CLD help both researchers and policy-makers to find the most important factors which have a huge potential to be considered in any decision so that they can overcome problems. It provides stakeholders with a comprehensive view on the issue of pharmaceutical abuse to take some steps to tackle the undesirable outcomes of such abnormal behavior in countries where the risk of abuse is high. Furthermore, although there are a wide range of studies that have identified variables affecting pharmaceutical abuse (11, 17, 33), they did not recognize the relationship among such variables. However, this study, via using CLD, contributes to depicting the complex relationships among such variables.
